# Determinants of breast cancer screening uptake in Kurdish women of Iran

**DOI:** 10.15171/hpp.2016.07

**Published:** 2016-03-31

**Authors:** Nayyereh Aminisani, Roujin Fattahpour, Saeed Dastgiri, Mohammad Asghari-Jafarabadi, Hamid Allahverdipour

**Affiliations:** ^1^Department of Statistics and Epidemiology, Tabriz University of Medical Sciences, Tabriz, Iran; ^2^Tabriz Health Services Management Research Centre, Tabriz University of Medical Sciences, Tabriz, Iran; ^3^Road Traffic Injury Research Center, Tabriz University of Medical Sciences, Tabriz, Iran; ^4^Clinical Psychiatry Research Center, Tabriz University of Medical Sciences, Tabriz, Iran

**Keywords:** Breast cancer screening, Socioeconomic status, Determinants, Minority, Iran

## Abstract

**Background: ** Recently, a national breast cancer screening program has been introduced in Iran.The aim of this study was to examine the determinants of breast cancer screening uptake among Kurdish women, in order to identify those characteristics that may be potentially associated with the screening uptake.

**Methods:** Through a cross sectional study, in 2014, a random sample of 561 women aged 40 years and older without the history of breast cancer and identified with Kurdish background in Baneh county, Iran, were recruited and interviewed by two trained interviewers. Data were collected using a valid and reliable researcher made questionnaire. Univariate and multivariate logistic regression models with self-reported screening history as the dependent variable were used to estimate the odds ratios (ORs) with 95% of CI.

**Results: ** The mean age of women was 43.64 (SD = 5.17). The participation rate in the mammography program was 16.8% (95% CI: 13.7-19.8%). The lowest level of participation was found among women aged 60 and older (OR = 0.30, 95% CI: 0.14-0.69), illiterate (OR = 0.63,95% CI: 0.40-0.99) and post-menopausal (OR = 0.56, 95% CI: 0.35-0.91) women.

**Conclusion:** It was found that the level of breast screening uptake was low among Kurdish women compared to those reported in the previous studies. Designing participation enhancing interventions with a specific focus on older, illiterate and post-menopausal women are recommended.

## Introduction

 Breast cancer is the most common cancer among women in both developed and less developed countries. In 2012, there had been reported about 1.67 million new cases of this cancer which comprised approximately 25.5% of all cancer cases.^1^ Globally, there is a regional variability in the incidence rates of this disease ranging from 27 per 100 000 in African and Middle Eastern countries to 96 per 100 000 in Western Europe and it is, also, the most frequent cause of cancer mortality among women in the less developed regions and the second in the developed countries.^1^

 Breast cancer mortality has fallen considerably after the introduction of breast cancer screening in the western countries.^2^ However, the screening is unavailable or less utilized (if available) in the developing countries where the majority of breast cancer deaths are occurred.^1^ Results from previous studies have shown that the pattern of breast cancer screening uptake is different among women throughout the world; women from socioeconomic disadvantaged groups, belong to ethnic or minority groups, disabled and frontiers have been reported to be in the least level of breast cancer screening participation rates.^3-7^

 In Iran, breast cancer is the most frequent cancer among women with incidence rate of 25 per 100 000.^8^ The National Breast Cancer Screening Program (NBCSP) was introduced in 2012 and is currently implemented in some cities with less than 50 000 inhabitants, to initial evaluation of the program, and will be expanded to all the cities in the future.^9^ According to its National Guideline, the following schedule for breast cancer screening is recommended: (1) Mammography: women older than 40 should have an annual mammogram, (2) Clinical breast examination (CBE): women with 20 to 40 years of age should have one CBE per 3 years and, also, after 40 an annual CBE by a health professional is emphasized, and (3) Breast self-examination (BSE): women older than 20 should perform monthly BSE.^9^

 Despite the potential benefits of mammography screening for breast cancer in women aged 40 and older, the findings of previous studies have revealed the low level of performance and ethnic variations in breast cancer screening among Iranian women (less than 1% to 44%).^10-15^

 Therefore, in the process of developing a nationwide screening program, it is important to identify the associated factors of breast cancer screening uptake in Iran, especially among women from minority groups living in frontiers. The findings may help the health policy makers in improving the NBCSP and achieving the goal of equity in health care services. Therefore, the aim of this study was to assess the level of breast cancer screening uptake among women aged 40 and older hoping to identify the mainly socio-demographic and health related determinants of participation in breast cancer screening in Baneh county, Iran.

## Materials and Methods

###  Participants and Procedures

 This cross-sectional study was conducted in Baneh county – a deprived area located in the west border of Iran with a total population of about 150 000 people. In 2014, stratified random sampling was employed to recruit 570 women aged 40 and older from six health centers to participate in the study. Inclusion criteria were aged 40 and older, being Iranian Kurd and with no history of breast cancer. They received a telephone invitation to attend a face to face interview in the health center, and if they were not able to come to the health center, they were suggested to be interviewed at home. In total, 561 women were accepted to participate in the study (participation rate = 98%).

###  Measures

 Data were collected using a researcher made structured questionnaire including three sections; (1) Demographic characteristics and socioeconomic status (SES), (2) Clinical and lifestyle characteristics including reproductive factors (parity, contraceptive [OCP] use, menopause status), family history of breast cancer, comorbidities, and overall health, and (3) Breast cancer screening (clinical examination and mammography).

 Age was classified into three categories: 40-49, 50-59, and 60+ years. Marital status was categorized as married (living with a spouse), and not married (divorced or widowed). SES was assessed using different variables including educational status, occupational status and insurance coverage. Educational status was categorized as illiterate and literate. Occupational status was, also, classified as in-paid work/retired and housewife. Family welfare was assessed by a question “How do you rate your family welfare?” with five Likert-type scale from “excellent to poor.” Comorbidities were based on the self-report history of frequent diseases like hypertension, diabetes, osteoporosis, heart diseases, and so on. Body mass index (BMI) was classified into three categories: healthy weight (BMI <25 kg/m^2^), overweight (BMI ≥25 kg/m^2^<30 kg/m^2^) and obese (BMI ≥30 kg/m^2^).

 The main outcome measures were having had the history of mammography by means of a mammogram and having had a history of mammography and/or clinical examination. The respondents were asked two questions regarding screening uptake: “Have you ever had a breast physical examination in which a medical doctor or health professional checked your breasts for lumps?” and “Have you ever had a mammography?” Having a mammogram and/or CBE (yes=1, no=0) were the dependent variables.

###  Statistical analysis

 Univariate analyses were used to describe the general characteristics of the study population. Multivariate logistic regression models with self-reported screening history (clinical examination/mammography) as the dependent variable were used to estimate odds ratios (ORs) with 95% of CI. Two models were built: adjusted for age, marital status, and education; and fully adjusted for age, marital status and education as well as health related factors (BMI, comorbidities, family history of breast cancer, and menopause status). Analysis was performed using SPSS version 23. Level of significance was considered to be 0.05, at the priori.

## Results

 A total of 561 women aged 40 and older were included in this study. Mean age of the women was 43.64 ± 5.17 and the majority (55%) was in the range of 40-49. The majority (81%) were married and currently live with their spouse, about 60% were illiterate and only 7% were involved in in-paid work. About 40% had no health insurance and 81% were overweight/obese. Also, 55.7% had at least a disease comorbidity which was mostly hypertension and diabetes. Less than 30% reported to be OCP users. About 12% had a family history of breast cancer and about 45% were in the postmenopausal status (Table 1).

**Table 1:** Characteristics among women 40 years and older in Baneh, west of Iran, 2014

**Variables**	**Number**	**Percent**
Age group		
40-49	305	54.5
50-59	149	26.6
60+	105	18.9
Marital status		
Married	453	80.9
Single/divorce/widow	107	19.1
Education		
Illiterate	329	58.6
Literate	232	41.4
Self-report family welfare		
Low	97	17.7
Middle	372	67.8
Upper middle and higher	79	14.5
Job status		
In-paid work/retired	39	07.0
Housewife	520	93.0
Insurance		
Yes	325	59.3
No	223	40.7
Comorbidities		
Yes	310	55.7
No	246	44.3
BMI		
Less than 25	101	18.9
25-25.9	229	42.5
30+	208	38.6
OCP use		
Yes	155	28.1
No	395	71.9
Menopause		
Yes	252	45.3
No	305	54.7
Family history		
Yes	66	11.8
No	490	88.2

 Mammography uptake was reported by 16.8% (n=90) of the women. Also, performing mammography and/or clinical breast screening were 22% (95% CI: 18.5-25.3%). Mammography uptake was less common among older women; crude ORs for having mammography were 0.86 (95% CI: 0.52-1.44) for women aged 50-59 and 0.30 (95% CI: 0.14-0.69) for women aged 60 and older in proportion to the younger women (40-49 years of age) (Table 2). Adjustment for age, marital status and educational status attenuated the OR for women aged 50-59 but had little effect for women aged 60 and older (OR=O.35, 95% CI: 0.15-0.84). Addition of some health-related factors (BMI, comorbidities, having the history of breast cancer, menopause status) into the model had more effect on the ORs especially among women aged 50-59; the effect increased as the uptake of mammography was 14% higher compared to the women aged 40-49, but it is still about 50% lower among women aged 60 and older in comparison with the younger women (OR=0.50, 95% CI: 0.17-1.48). Crude OR for women who live with their spouse was 0.80 (95% CI: 0.46-1.38) in proportion to the single/divorced/widowed women. Adjustment in both models had very little effect on ORs.

**Table 2:** Mammography uptake by demographic & socioeconomic factors, and health related factors among women 40 years and older in Baneh, west of Iran, 2014

	**Mammography**				
**Variable**	**Total**	**Uptake N (%)**	**OR** ^a^ ** (CI 95%)**	**OR** ^b^ ** (CI 95%)**	**OR** ^c^ ** (CI 95%)**
Age group					
40-49	305	58 (19.01)	Referent	Referent	Referent
50-59	149	25 (16.77)	0.86 (0.52-1.44)	0.93 (0.54-1.63)	1.14 (0.56-2.34)
60+	105	7 (6.60)	0.30 (0.14-0.69)	0.35 (0.15-0.84)	0.50 (0.17-1.48)
*P* value			0.02	0.06	0.22
Marital status					
Married	453	70 (15.45)	0.80 (0.46-1.38)	0.81 (0.47-1.41)	0.77 (0.44-1.38)
Single/divorce/widow	107	20 (18.69)	Referent	Referent	Referent
*P* value			0.41	0.46	0.38
Education					
Illiterate	328	44 (13.41)	0.63 (0.40-0.99)	0.81 (0.49-1.34)	0.82 (0.48-1.40)
literate	232	46 (19.82)	Referent	Referent	Referent
*P* value			0.04	0.41	0.46
Comorbidities					
Yes	310	44 (14.19)	0.72 (0.46-1.13)	0.86 (0.54-1.38)	0. 89 (0.54-1.46)
No	246	46 (18.69)	Referent	Referent	Referent
*P* value			0.15	0.53	0.64
BMI					
Less than 25	101	17 (16.83)	Referent	Referent	Referent
25-29.9	229	37 (16.15)	0.95 (0.51-1.79)	0.84 (0.44-1.59)	0.87 (0.45-1.66)
30+	208	34 (16.34)	0.97 (0.51-1.83)	0.84 (0.44-1.61)	0. 84 (0.44-1.63)
*P* value			0.99	0.84	0.87
Menopause					
Yes	252	30 (11.9)	0.56 (0.35-0.91)	0.72 (0.36-1.45)	0.76 (0.37-1.59)
No	305	59 (19.34)	Referent	Referent	Referent
*P* value			0.02	0.31	0.47
Family history					
Yes	66	13 (19.69)	1.34 (0.70-2.57)	1.32 (0.68-2.56)	1.35 (0.69-2.63)
No	490	76 (15.51)	Referent	Referent	Referent
*P* value			0.39	0.41	0.39

Numbers obtained by adding together categories for each variable may not always add to total sample size due to missing data ^a^Crude odds ratio; ^b^Odds ratio adjusted for age, marital status, and education; ^c^Odds ratio adjusted for age, marital status, education, BMI, comorbidities, and history of breast cancer, menopause status

 The odds of breast cancer screening uptake were less common among illiterate compared to the literate women (OR=0.63, 95% CI: 0.40-0.99). Adjustment for age and marital status and health-related factors had little effect on the ORs of being screened among illiterate women. Among women with comorbidities, ORs were lower compared to the women with no comorbid disease (crude OR=0.72, 95% CI: 0.46-1.13), and there was found an increase in ORs (fully adjusted OR=0.89, 95% CI: 0.54-1.46), after adjustment. Mammography uptake was similar according to BMI categories, however, after adjustment, there was a decrease in ORs (both adjusted models) among women with BMI ≥30 (Fully adjusted OR=0.84, 95% CI: 0.44-1.63). In proportion to the women in pre-menopause category, post-menopausal women were less likely to have performed mammography (crude OR=0.56, 95% CI: 0.35-0.91) but the effect was attenuated a little (fully adjusted OR=0.76, 95% CI 0.37-1.59), after adjustment, and was no longer statistically significant. Women with a family history of breast cancer were 34% more likely to report mammography compared to the women without family history, and, fully adjustment did not change the effect.

## Discussion

 This study aimed to examine breast cancer screening uptake and its determinants in a minority group in Iran; Kurdish women living in Baneh county – a deprived area in the west of country. Iran is in the stage of implementing NBCSP which must cover women from diverse ethnic backgrounds, different SES and wide geographical areas. Therefore it is important to identify potential determinants of screening uptake.

 The overall uptake of mammography in this study was about 17% which is lower than those reported in developed countries; UK: 77%, USA: 67%, Finland: 87%, and, Italy: 60%^16-18^ but it is somewhat more common among those reported Turkmen women (less than 1%)^10^ followed by women from south east of Iran^11^ which was 1.5%. The highest uptake of mammography reported from Isfahan (44.3%).^12^ Lack of access to mammography facilities in this county might be an explanation for the low screening uptake, since the nearest mammography center requires one hour driving.

 It was found that women in the younger (40-49) and older (60+) ages had the higher and the lower percentages of mammography uptake, respectively. Moreover, women with the postmenopausal status were less likely to report the mammography screening uptake. It is similar to the findings of previous studies in other countries^19^ and in Iran,^12^ as well. Whilst older women are more likely to develop breast cancer and should be targeted for health promotion program.

 In the present study, mammography uptake was higher among literate women. Those with no health insurance had a lower percentage of uptakes. Many previous studies have found that women in socioeconomically disadvantaged groups had a lower participation rate in breast cancer screening,^3,6,7,20^ for example, results of an Italian study^21^ showed that women with higher levels of education were more likely to have a mammogram than those with a lower level (OR=1.28, 95% CI: 1.10-1.49) and women of intermediate and high occupational classes were more likely to use breast cancer screening (OR=1.77, 95% CI: 1.55-2.03; OR=1.63, 95% CI: 1.40-1.91) compared to unemployed women.

 The results showed that women with comorbidities had lower screening uptake compared to those without comorbidities which was attenuated after fully adjustment. However, a previous study^22^ reported that comorbid condition is associated with a regular mammography and earlier stage of diagnosis. This is because women who receive comorbidity-related care are more likely to receive regular mammography as a result of regular primary care visits. In contrast, another study^23^ reported the lower timely mammography among women of different racial/ethnical groups who had comorbidity because they might receive underserved medical care.

 In the present study, it was found that undergoing mammography was more common among women with normal weight compared to the overweight/obese women, but after adjustment, overweight/ obese women had a lower uptake compared to women with normal weight. The similar results were reported in Korean^24^ and German studies.^25^ However, in another study by Tekkel et al,^26^ in Estonia, opposite findings were reported. This might be explained in a way that obese women are less likely to adhere screening recommendation, or use the health services in the host countries.

 In this study, mammography use was high among women with a family history of breast cancer. The same results were reported by another study.^27^ This might be explained by the fact that such women have higher perceived susceptibility and or severity on breast cancer. Another possibility is that the physicians might be more sensitive about these women and prescribe them for mammography.

 There were some strengths and limitations for the present study. It was the first study to examine the mammography screening uptake among Kurdish women aged 40 years and older living in frontiers where the facilities for doing screening is poor. However, the overall low uptake (16%) might be influenced by some other determinants that were not studied in the present research.

## Conclusion

 The results of this study indicated that the mammography uptake among Kurdish women in Baneh, is low, and highlighted the need for implementing a comprehensive educational intervention, which should be considered as a top priority for health policy makers and providers. Also, the lack of mammography facilities nearby might be an important barrier and needs to be considered in the process of developing the NBCSP as the main infrastructure. In the present study, some influential factors were identified which may help to design and deliver an appropriate educational intervention to the target population. Further research is recommended to find out the potential barriers of screening participation in such areas.

## Ethical approval

 This study received ethical approval from Ethics Committee in Tabriz University of Medical Sciences. All participants completed a consent form before the interviews.

## Competing interests

 The authors have no conflicts of interest to declare.

## Acknowledgements

 This study was funded by the research council, Tabriz University of Medical Sciences for which we are thankful. We also extend our gratitude to the head of Baneh Health Centre, and those who contributed to data collection and all women who participated in this study.
